# Supplementary material for: Anabasis articulata (Forssk.) Moq: A Good Source of Phytochemicals with Antibacterial, Antioxidant, and Antidiabetic Potential
Source: Molecules. 2022 May 30;27(11):3526. doi: 10.3390/molecules27113526 (PMC9182432; doi:10.3390/molecules27113526)
Supplement: Supplementary file 1 [file molecules-27-03526-s001.zip › molecules-1742475-supplementary.pdf]

molecules

Article

Anabasis articulata (Forssk.) Moq: A Good Source of Phytochemicals with Antibacterial, Antioxidant, and Antidiabetic Potential

Fakhria A. Al-Joufi [1], Marwa Jan [2], Muhammad Zahoor [2,*], Nausheen Nazir [2], Sumaira Naz [2], Muhammad Talha [2], Abdul Sadiq [3], Asif Nawaz [3] and Farhat Ali Khan [4]

[1] Department of Pharmacology, College of Pharmacy, Jouf University, Aljouf 72341, Saudi Arabia; faaljoufi@ju.edu.sa
[2] Department of Biochemistry, University of Malakand, Chakdara, Dir Lower 18800, KPK, Pakistan; jmarwa084@gmail.com (M.J.); nausheen.nazir@uom.edu.pk (N.N.); sumaira.biochem@gmail.com (S.N.); livingontheedge36837@gmail.com (M.T.)
[3] Department of Pharmacy, University of Malakand, Chakdara, Dir Lower 18800, KPK, Pakistan; sadiquom@yahoo.com (A.S.); asifnawaz2445@gmai.com (A.N.)
[4] Department of Pharmacy, Shaheed Benazir Bhutto University, Sheringal, Dir Upper 18050, KPK, Pakistan; farhatkhan2k9@yahoo.com
* Correspondence: mohammadzahoorus@yahoo.com or zahoor@uom.edu.pk

Supplementary File

Table S1. Major phytochemical compounds identified in fraction A of *A. articulata* and their various parameters.

Retention time (min)	Molecular mass (g/mol)	Molecular formula	%Area	Area	Peak height	Compound name
3.18	87	C_4H_9NO	0.34	43384913 .99	3301173.37	Acetdimethylamide
6.13	116	$C_4H_8N_2O_2$	0.00	416041.0	142054.22	N-Nitrosomorpholine
22.80	390	$C_{24}H_{38}O_4$	0.01	1071457. 11	486644.85	1,2-Benzenedicarboxylic acid
22.80	278	$C_{16}H_{22}O_4$	0.01	1071457. 11	486644.85	Mono(2-ethylhexyl) phthalate
22.80	390	$C_{24}H_{38}O_4$	0.01	1071457. 11	486644.85	Bis(2-ethylhexyl) phthalate

Table S2. Major phytochemical compounds and their parameters identified in fraction B of *A. articulata.*

Retention time (min)	Molecular mass (g/mol)	Molecular formula	Area %	Area	Peak height	Compound name
6.15	190	$C_7H_{14}N_2O_2S$	0.00	545346.31	147100.04	N-Acetyl-l-methioninamide
6.15	116	$C_5H_{12}N_2O$	0.00	545346.31	147100.04	2-Propanamine
13.22	206	$C_{14}H_{22}O$	0.01	1244394. 35	419011.73	Phenol, 2,4-di-tert-butyl
18.01	428	$C_{31}H_{56}$	0.00	333580.17	201943.96	Benzene, (1-dodecyltridecyl)-
18.01	358	$C_{26}H_{46}$	0.00	333580.17	201943.96	Benzene, (1-hexyltetradecyl)-
18.01	260	$C_{19}H_{32}$	0.00	333580.17	201943.96	Benzene, (1-hexylheptyl)-
19.66	298	$C_{19}H_{38}O_2$	0.01	1020939. 00	571661.96	Isopropyl Palmitate

20.21	296	$C_{19}H_{36}O_2$	0.01	1136112. 80	346764.23	10-Octadecenoic acid, methyl ester
20.78	308	$C_{22}H_{44}$	0.01	1106327. 24	545734.08	1-Docosene
21.45	312	$C_{19}H_{36}O_3$	0.04	5008111. 95	581962.66	Methyl ricinoleate
22.29	478	$C_{32}H_{62}O_2$	0.00	394588.26	205719.11	Oleic acid, tetradecyl ester
22.82	390	$C_{24}H_{38}O_4$	0.10	13672399 .66	13672399.66	Diisooctyl phthalate

Table S3. List of major components and their parameters identified in fraction C of *A. articulata.*

Retention time (RT)	Molecular mass (g/mol)	Molecular formula	Area %	Area	Peak height	Compound name
3.28	132	C4H8N2O3	0.12	9646066.72	920854.34	Asparagine
18.09	428	C31H56	0.01	680297.28	303315.64	entacosane, 13-phenyl
18.09	358	C26H46	0.01	680297.28	303315.64	Eicosane, 7-phenyl
18.09	246	C18H30	0.01	680297.28	303315.64	Dodecane, 6-phenyl
18.99	270	C17H34O2	0.01	680297.28	303315.64	Palmitic acid, methyl ester
19.74	298	C19H38O2	0.02	1900738.29	1069011.97	Isopropyl Palmitate
21.19	258	C16H34S	0.01	588569.28	284675.46	tert-Hexadecanethiol
21.49	312	C19H36O3	0.06	4798752.56	1232061.04	Methyl ricinoleate
22.25	422	C28H54O2	0.01	704407.97	357520.57	Decyl oleate
22.25	534	C36H70O2	0.01	704407.97	357520.57	Oleic acid, octadecyl ester
22.25	324	C21H40O2	0.01	704407.97	357520.57	Elaidic acid, isopropyl ester
22.88	390	C24H38O4	0.11	8499930.38	3479760.69	Diisooctyl phthalate
22.88	278	C16H22O4	0.11	8499930.38	3479760.69	Mono(2-ethylhexyl) phthalate

Table S4. Major phytochemical compounds and their parameters identified in fraction D of *A. articulata.*

Retention time (RT)	Molecular mass (g/mol)	Molecular formula	Area %	Area	Peak height	Compound name
9.17	136	$C_9H_{12}O$	0.44	2730076. 55	486447.80	Phenethyl alcohol, á-methyl
11.98	177	$C_{11}H_{15}NO$	0.20	1259580. 89	468584.80	Benzyl-3-hydroxypyrrolidine
14.89	222	$C_{12}H_{14}O_4$	0.17	1068281. 67	278449.71	Diethyl Phthalate
15.79	223	$C_9H_{13}N_5O_2$	1.83	11259232 .47	2473842.30	2,6-Dimethyl-pyridine-3,5-dicarboxylic acid, dihydrazide
15.79	223	$C_{10}H_9NO_3S$	1.83	11259232 .47	2473842.30	Methoxycarbonyl-2-methoxyphenyl isothiocyanate
16.12	348	$C_{14}H_{28}F_3O_4P$	0.10	594434.25	151973.27	Phosphoric acid, dibutyl 3-trifluoromethyl-3-pentyl ester
16.40	282	$C_{18}H_{34}O_2$	0.03	213490.32	121076.06	Oleic Acid

17.77	251	$C_{12}H_{13}NO_5$	0.25		371405.90	4-Acetylaminophthalic acid, dimethyl ester
17.77	251	$C_{12}H1_3NO_5$	0.25	1510981. 99	371405.90	Benzene-1,3-dicarboxylic acid, 5-acetylamino-, dimethyl ester
18.15	444	$C_{28}H_{44}O_4$	0.51	3167087. 78	1195028.44	(2-Phenyl-1,3-dioxolan-4-yl) methyl (9E)-9-octadecenoate
20.90	340	$C_{22}H_{44}O_2$	0.32	1968742. 62	729758.88	1-Heneicosyl formate
22.70	352	$C_{21}H_{24}N_2O_3$	0.30	1828910. 15	327967.98	18,19-Secoyohimban-19-oic acid,
22.70	340	$C_{21}H_{28}N_2O_2$	0.30	1828910. 15	327967.98	Cleavamine, 18á-carboxy-3,4à-dihydro-, methyl ester

Table S5. Major phytochemical compounds and their parameters identified in the oil fraction of *A. articulata.*

Retention time (RT)	Molecular mass (g/mol)	Molecular formula	Area %	Area	Peak height	Compound name
7.42	113	$C_6H_{11}N_O$	0.01	851036. 36	308481.86	1-Piperidinecarboxaldehyde
7.42	113	$C_7H_{15}N$	0.01	1851036. 36	308481.86	(1-Ethyl-propenyl)-dimethyl-amine

Table S6. Antibacterial potential of crude extract, oil, and purified fraction of *A. articulata.*

Microbial strain	%Zone of Inhibition (mm)						
	Crude extract	Oil Fraction	Fraction A	Fraction B	Fraction C	Fraction D	Ampicillin (positive control)
Escherichia coli	11±0.5***	24±1.3***	15±0.3***	11±1.3***	18±2.5***	11±0.9***	34±0.3
Shigella dysentery	19±1.0***	20±1.8***	16±0.9***	0	16±0.4***	19±1.2***	32±0.6
Salmonella typhi	10±2.0***	16±2.3***	19±1.5***	0	0	16±2.0***	30±0.2

The data is represented as Mean ± SEM, n=3. Values are significantly different as compared to positive control *$^{***}P< 0.001$.*

Table S7. Antioxidant potential (DPPH and ABTS) of *A.articulata* crude extract, hexane, and subsequent fractions.

Sample	Concentration (μg/mL)	%DPPH Scavenging	IC_{50} (μg/mL)	%ABTS Scavenging	IC_{50} (μg/mL)
Crude extract	1000	88.45±0.97**	90	84.35±0.86***	50
	500	73.68±0.71**		75.58±0.84***	
	250	66.93±0.74***		63.91±0.57***	
	125	58.48±0.63***		59.53±0.37**	
	62.5	47.75±0.56***		52.69±0.78*	
Fraction A	1000	73.69±0.32***	200	86.48±0.85***	75
	500	71.31±0.45***		78.79±0.81***	
	250	69.20±0.12***		72.45±0.79**	
	125	62.38±0.14**		63.47±0.67*	
	62.5	53.61±0.21***		49.89±0.47**	
Fraction B	1000	82.60±0.43***	62	75.49±0.57***	110
	500	78.90±0.35***		66.90±0.38***	
	250	71.34±0.65**		59.48±0.27***	
	125	57.16±0.56***		51.38±0.42***	
	62.5	50.12±0.55***		43.95±0.37***	
Fraction C	1000	42.80±0.54***	1200	79.59±0.39***	118

	500	34.11±0.87***		71.48±0.62***	
	250	30.41±0.83***		64.78±0.69***	
	125	24.88±0.35***		57.74±0.48***	
	62.5	21.56±0.67***		48.37±0.68***	
Fraction D	1000	53.08±0.76***	250	85.95±0.61***	99
	500	52.34±0.24***		74.64±0.58***	
	250	50.36±0.88***		67.86±0.95***	
	125	49.77±0.85***		60.29±0.82**	
	62.5	47.88±0.63***		55.35±0.91**	
Oil Fraction	1000	91.43±0.88^{ns}	45	82.17±0.84***	71
	500	84.65±0.65*		79.41±0.43**	
	250	78.24±0.32^{ns}		64.52±0.39***	
	125	60.56±0.54**		58.58±0.23***	
	62.5	56.37±0.98**		50.54±0.73**	
Ascorbic acid	1000	94.88±0.96	30	91.11±0.68	32
	500	86.59±0.81		83.42±0.43	
	250	78.64±0.63		78.35±0.73	
	125	69.14±0.34		65.15±0.61	
	62.5	62.87±0.45		57.14±0.33	

The data is represented as Mean ± SEM, n=3. Values are significantly different as compared to positive control **P*< 0.05, ***P*< 0.01, ****P*< 0.001.*

Table S8 α- glucosidase and α- amylase inhibition of *A. articulata* crude extract and subsequent purified fractions at various concentrations.

**Sample**	**Concentration (μg/mL)**	**%α-Glucosidase inhibition**	**IC$_{50}$ (μg/mL)**	**% α-Amylase inhibition**	**IC$_{50}$ (μg/mL)**
		Mean ±SEM		Mean ±SEM	
Met- Ext	1000	85.32±0.20**	32	83.98±0.21*	34
	500	81.89±0.09^{ns}		81.29±0.08*	
	250	70.25±0.65**		70.32±0.34**	
	125	67.45±0.34*		65.38±0.36**	
	62.5	68.23±1.34*		62.28±0.67*	
Fraction A	1000	62.15±0.84***	200	59.34±0.89***	240
	500	58.92±0.98***		52.67±0.16***	
	250	51.08±0.64***		49.32±0.23***	
	125	46.18±0.56***		43.65±0.56***	
	62.5	43.25±1.78***		32.78±0.78***	
Fraction B	1000	78.51±0.45***	60	81.32±0.09**	58
	500	70.32±0.73***		78.61±0.30***	
	250	65.57±0.03***		62.56±0.13***	
	125	59.28±1.03***		59.18±0.07***	
	62.5	53.58±1.08***		52.98±0.15***	
Fraction C	1000	72.57±0.92***	120	74.52±0.13***	180
	500	69.52±0.45***		69.88±0.71***	
	250	55.88±0.18***		61.65±0.23***	
	125	51.78±0.15***		57.32±0.91***	
	62.5	47.31±1.02***		50.54±0.37***	

Fraction D	1000	70.61±0.91***	140	72.51±0.02***	200
	500	63.59±0.08***		69.41±0.04***	
	250	54.12±0.67***		59.91±0.38***	
	125	49.04±0.71***		51.31±0.15***	
	62.5	43.25±0.90***		47.18±0.76***	
Oil Fraction	1000	61.14±0.49***	180	58.14±0.75***	220
	500	58.82±0.19***		53.61±0.92***	
	250	50.08±0.04***		48.22±0.49***	
	125	45.28±0.92***		40.55±0.07***	
	62.5	41.34±0.28***		37.18±0.16***	
Acarbose	1000	87.65±0.71		85.99±0.44	
	500	83.05±0.65	30	83.61±0.58	32
	250	78.90±1.02		76.85±0.96	
	125	71.83±0.99		70.47±0.78	
	62.5	65.15±0.75		64.89±0.71	

The data is represented as Mean ± SEM, n=3. Values are significantly different as compared to positive control **P< 0.05, **P< 0.01, ***P< 0.001*.

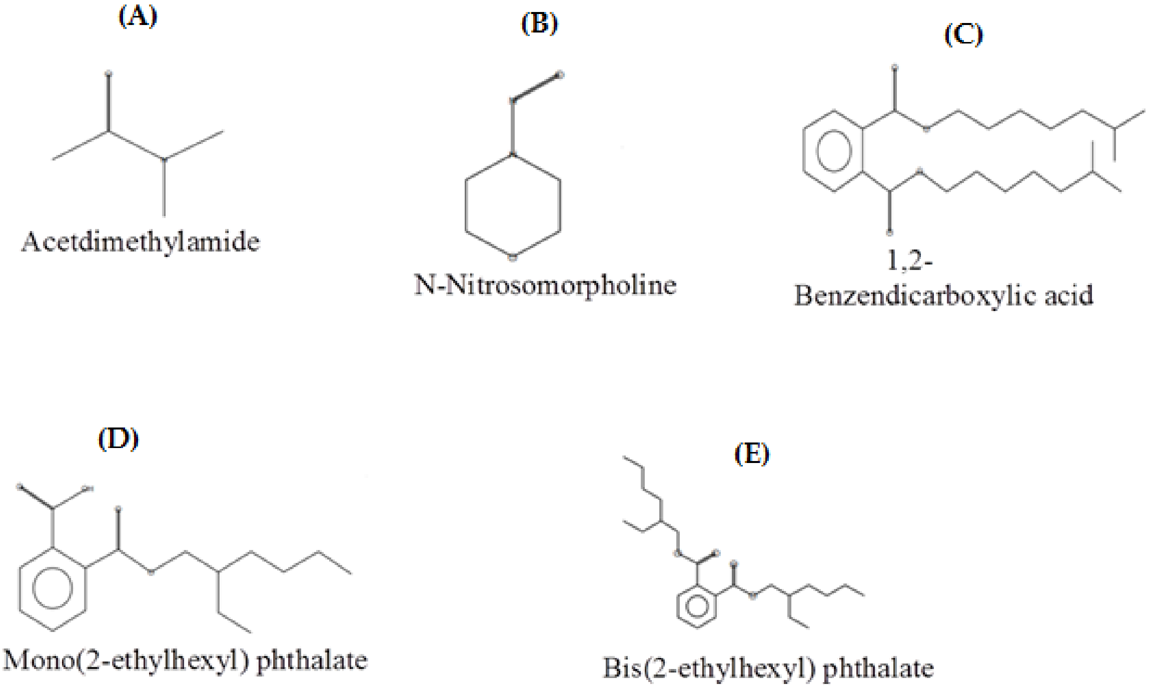

Figure S1. Major phytochemical compounds identified in fraction A of *A. articulata*.

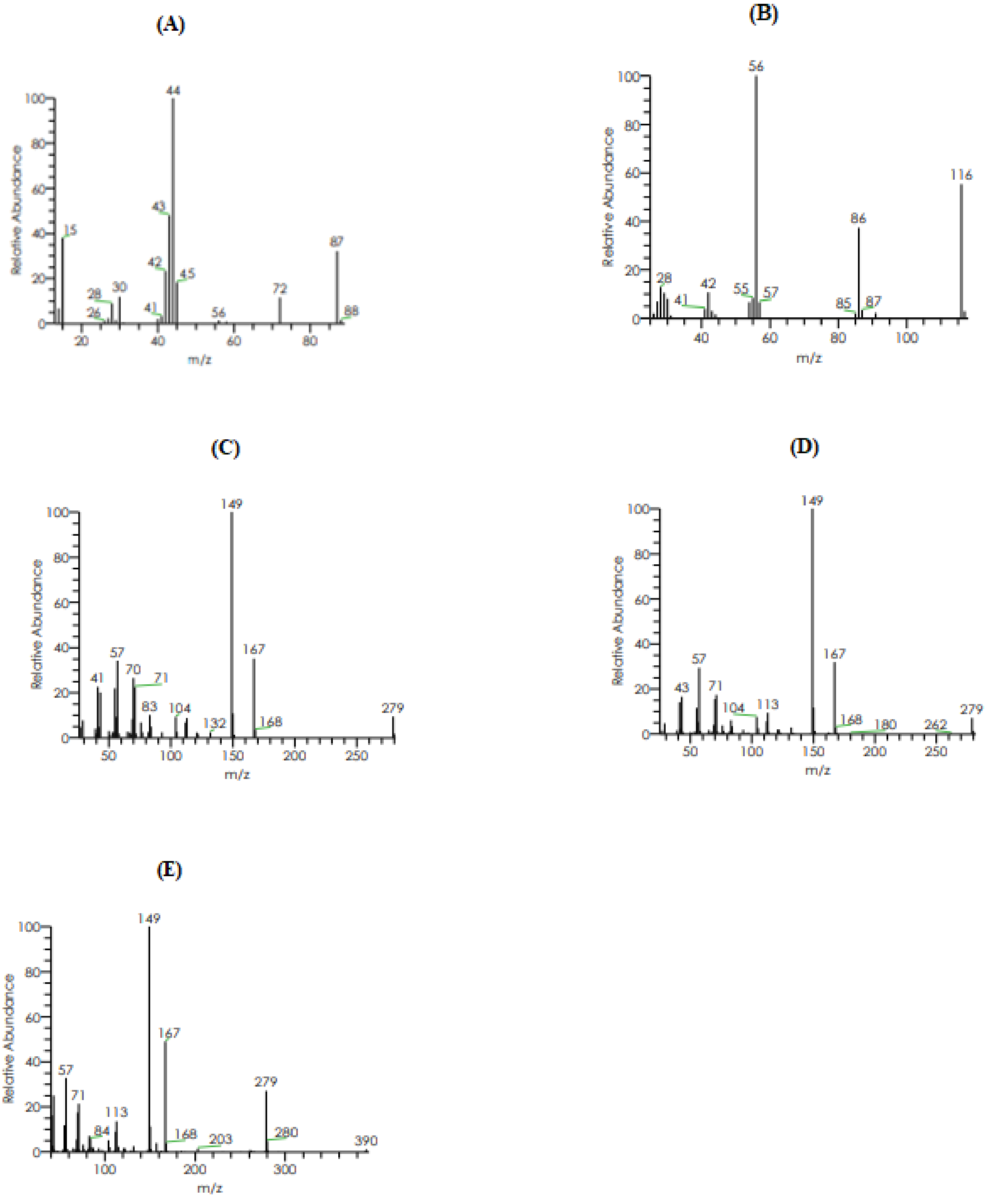

Figure S2. Individual mass fragmentation patterns of each compound: **A)** Acetdimethylamide **B)** N-Nitrosomorpholine **C)** 1,2-Benzenedicarboxylic acid **D)** Mono(2-ethylhexyl) phthalate **E)** Bis(2-ethylhexyl) phthalate.

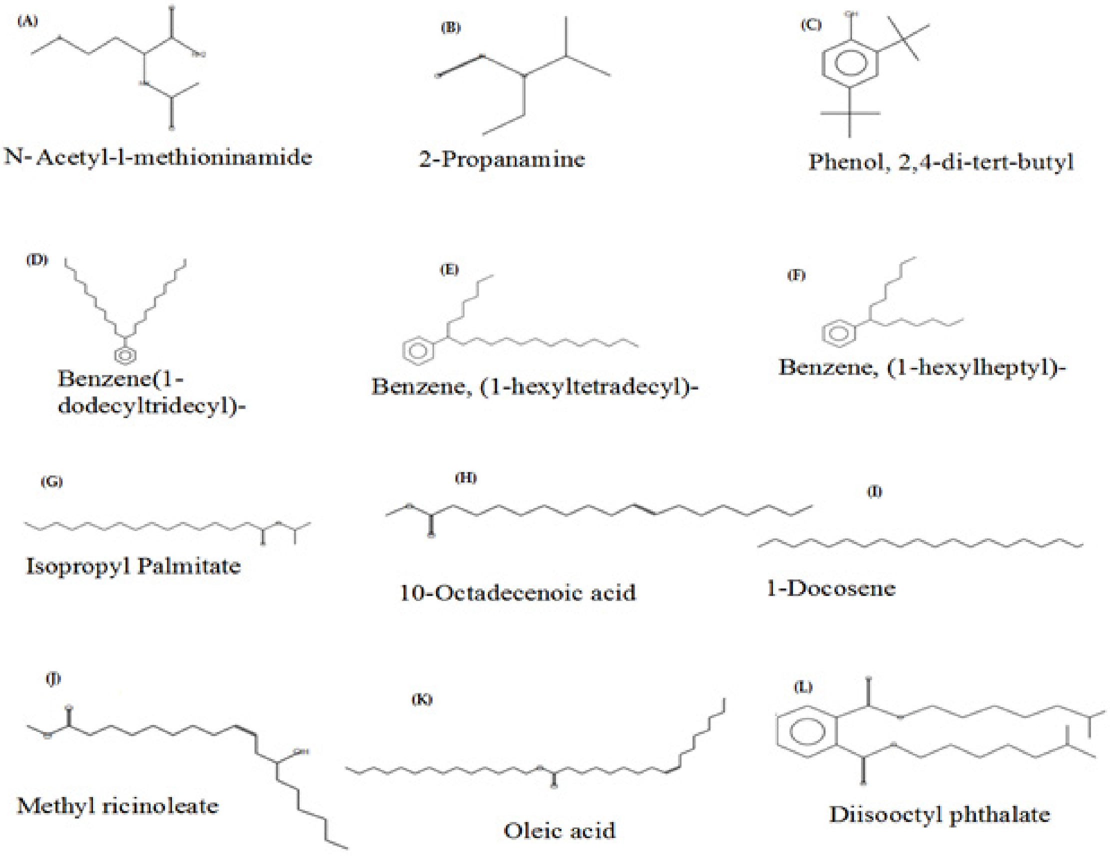

Figure S3. Major phytochemical compounds identified in fraction B of *A. articulata.*

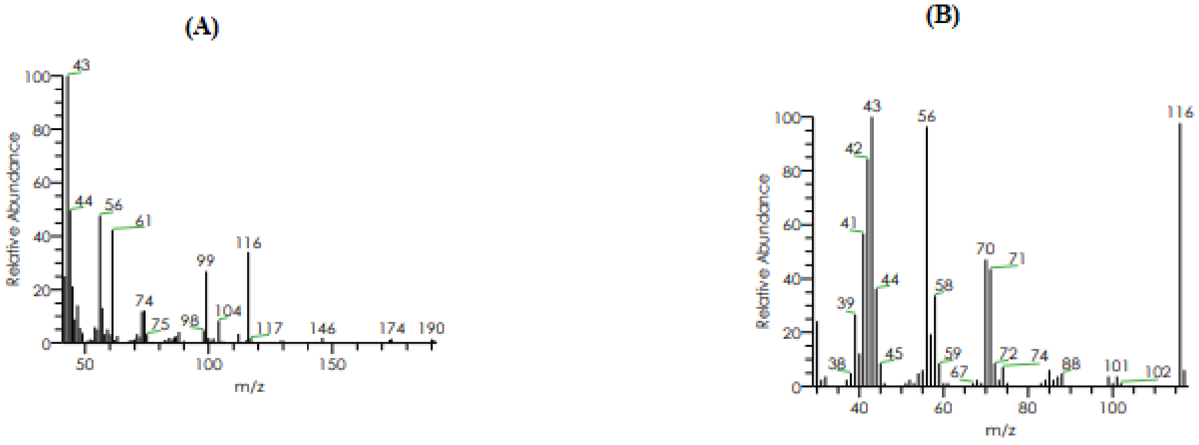

(C)

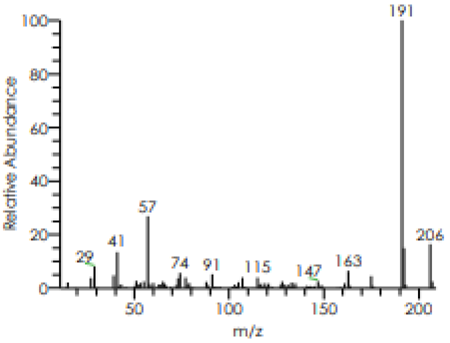
Relative Abundance
m/z
191
206
57
41
29
74
91
115
147
163

(D)

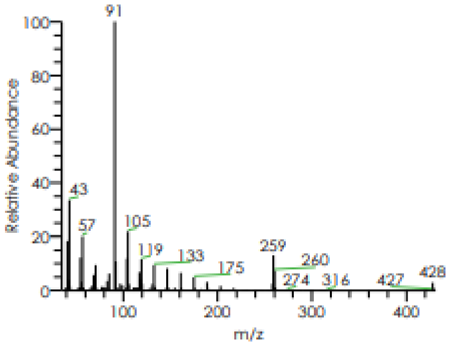
Relative Abundance
m/z
91
43
57
105
119
133
175
259
260
274
316
427
428

(E)

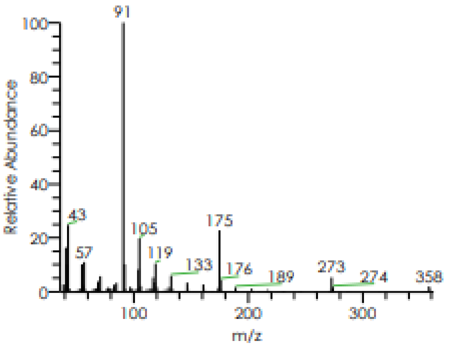
Relative Abundance
m/z
91
43
57
105
119
133
175
176
189
273
274
358

(F)

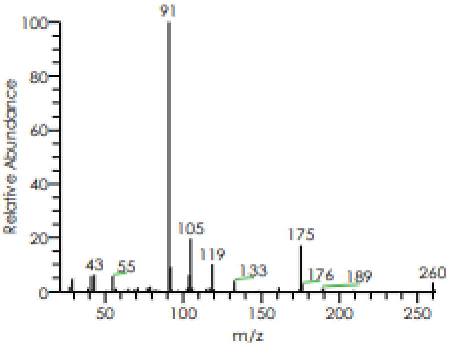
Relative Abundance
m/z
91
43
55
105
119
133
175
176
189
260

(G)

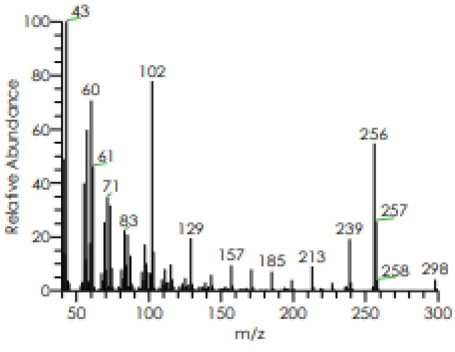
Relative Abundance
m/z
43
60
61
71
83
102
129
157
185
213
239
256
257
258
298

(H)

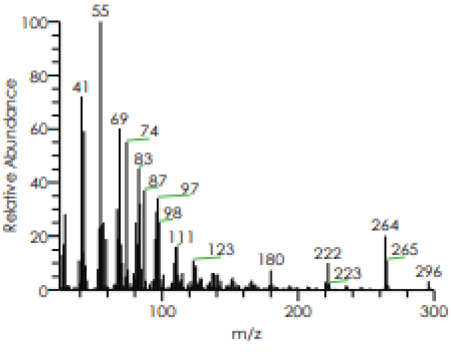
Relative Abundance
m/z
55
41
69
74
83
87
97
98
111
123
180
222
223
264
265
296

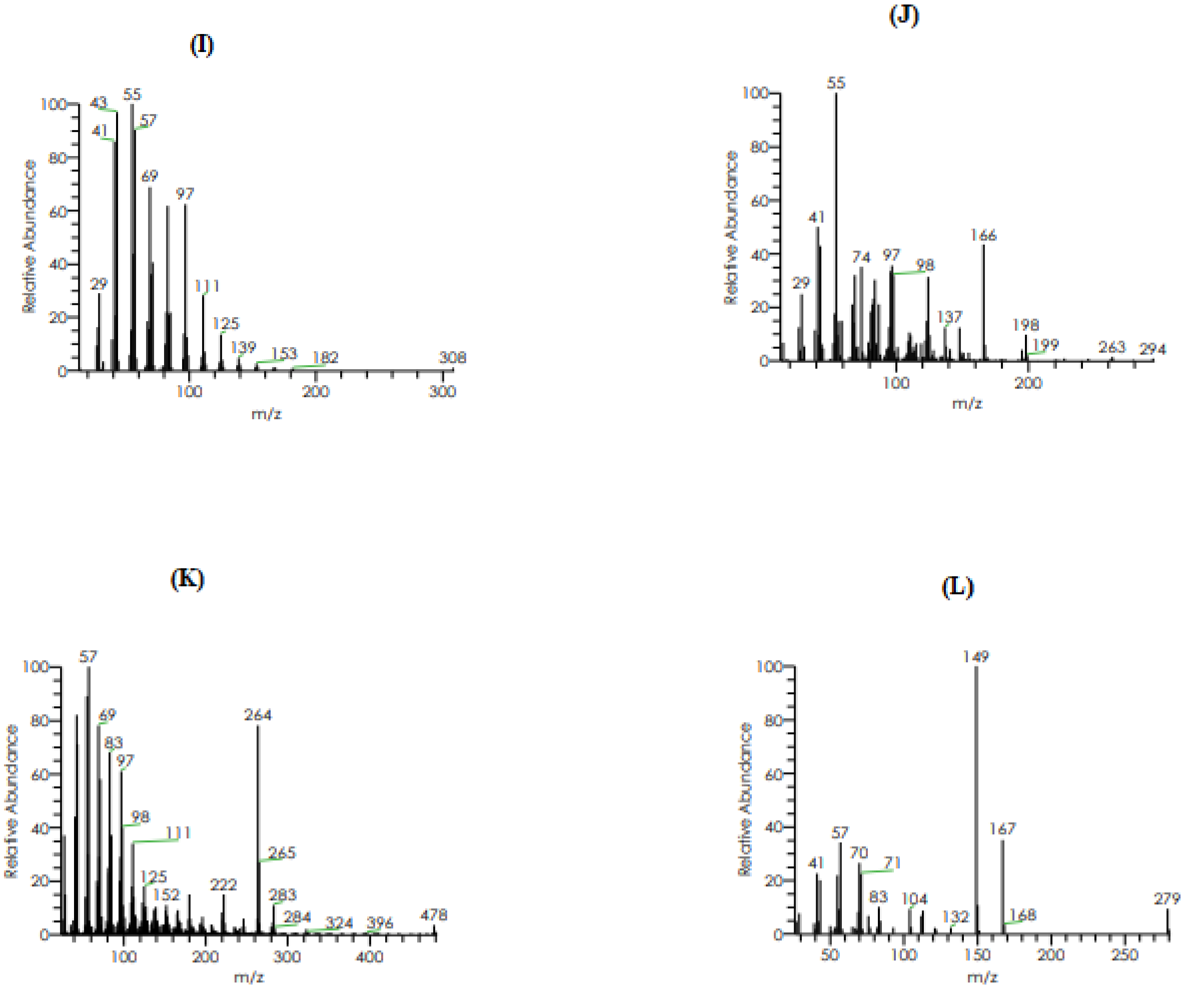

Figure S4. Individual mass fragmentation patterns of each compound: **A)** N-Acetyl-l-methioninamide **B)** 2-Propanamine **C)** Phenol, 2,4-di-tert-butyl **D)** Benzene, (1-dodecyltridecyl) **E)** Benzene, (1-hexyltetradecyl) **F)** Benzene, (1-hexylheptyl **G)** Isopropyl Palmitate **H)** 10-Octadecenoic acid, methyl ester **I)** 1-Docosene **J)** Methyl ricinoleate **K)** Oleic acid, tetradecyl ester **L)** Diisooctyl phthalate.

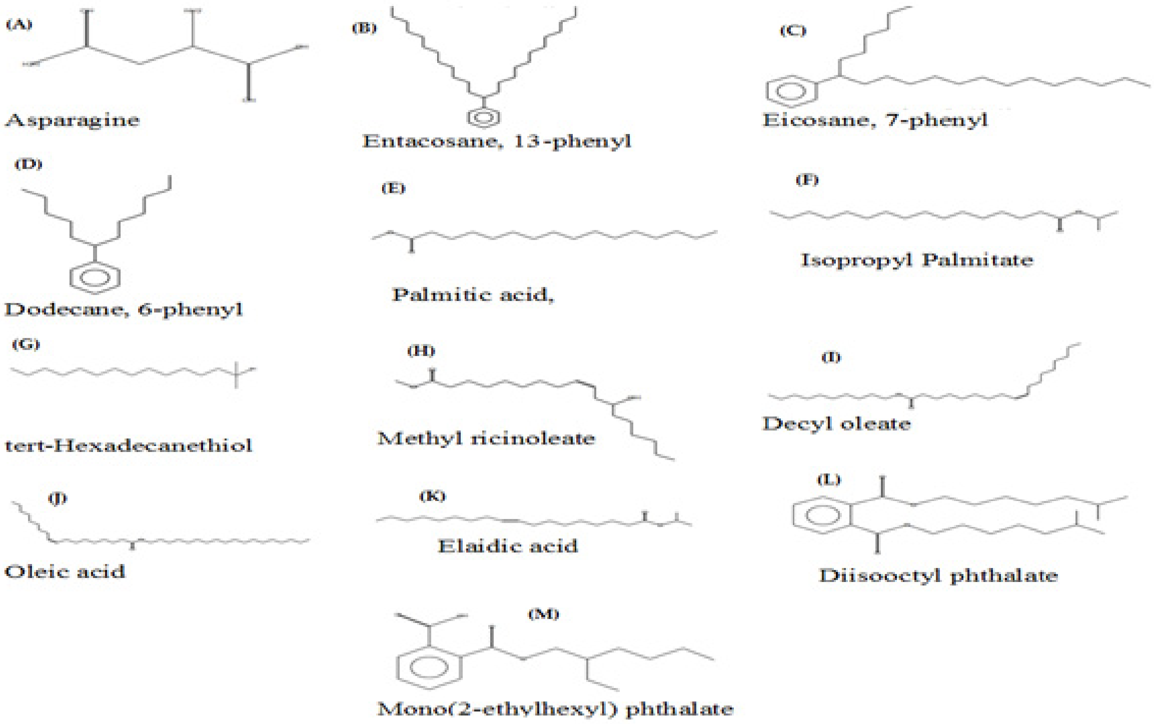

Figure S5. Major phytochemical compounds identified in fraction C of *A. articulata*.

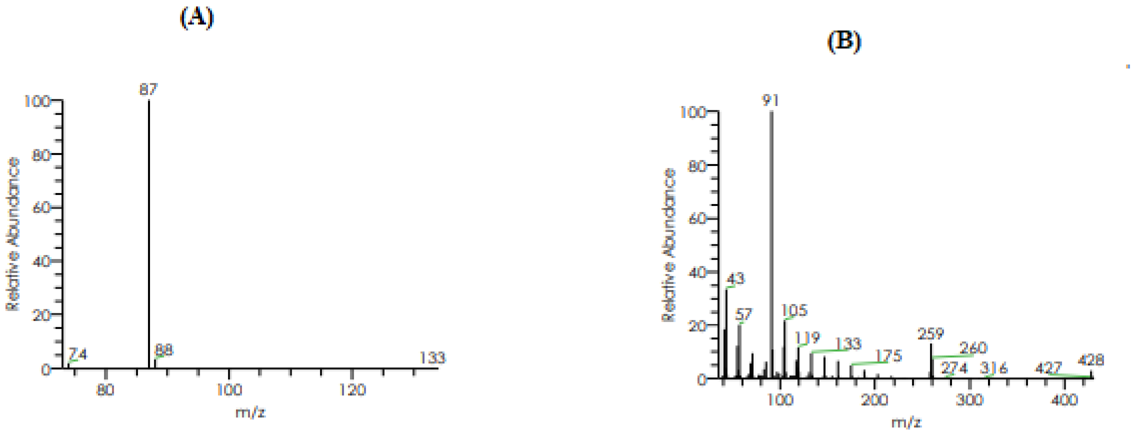

(C)

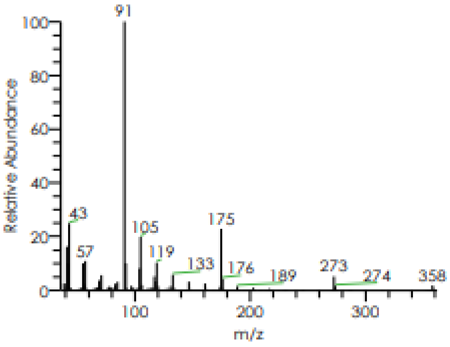
Relative Abundance
m/z
91
43
105
175
57
119
133
176
189
273
274
358

(D)

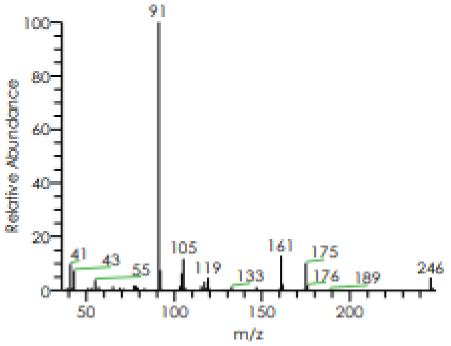
Relative Abundance
m/z
91
41
43
55
105
119
133
161
175
176
189
246

(E)

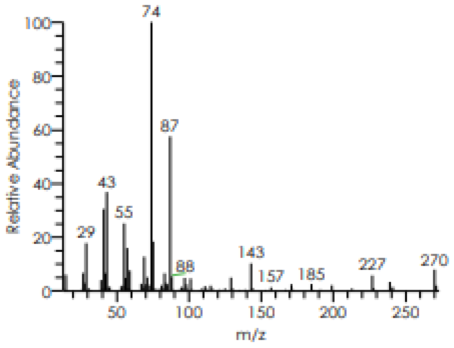
Relative Abundance
m/z
74
87
43
55
29
88
143
157
185
227
270

(F)

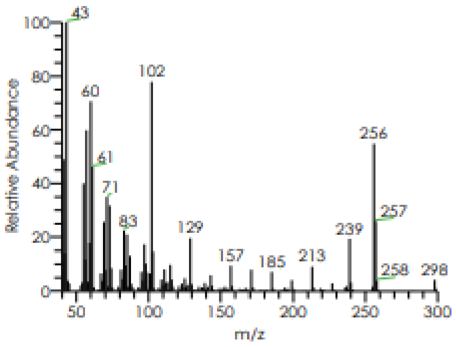
Relative Abundance
m/z
43
102
60
256
61
71
83
129
257
239
157
185
213
258
298

(G)

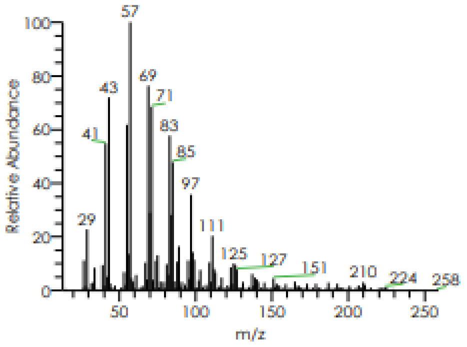
Relative Abundance
m/z
57
69
43
71
83
41
85
97
29
111
125
127
151
210
224
258

(H)

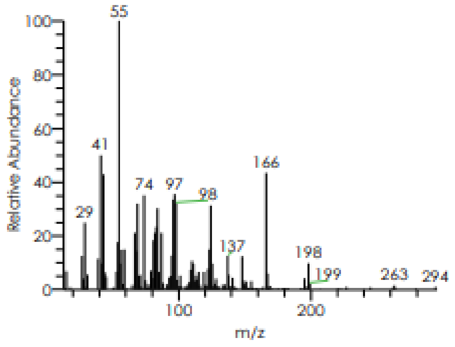
Relative Abundance
m/z
55
41
166
74
97
98
29
137
198
199
263
294

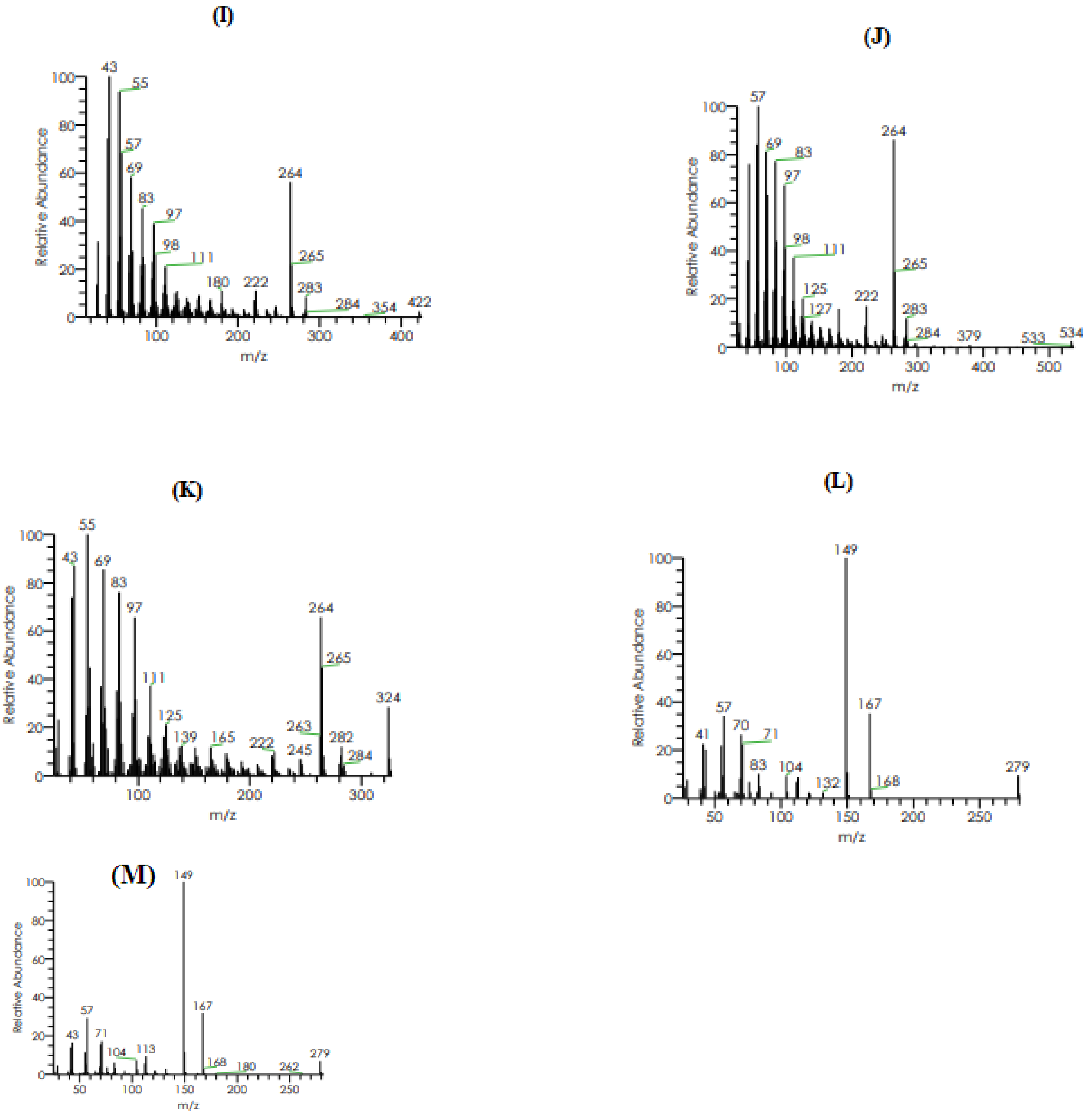

Figure S6. Individual mass fragmentation patterns of each compound: **A)** Asparagine **B)** entacosane, 13-phenyl **C)** Eicosane, 7-phenyl **D)** Dodecane, 6-phenyl **E)** Palmitic acid, methyl ester **F)** Isopropyl Palmitate **G)** tert-Hexadecanethiol **H)** Methyl ricinoleate **I)** Decyl oleate **J)** Oleic acid, octadecyl ester **K)** Elaidic acid, isopropyl ester **L)** Diisooctyl phthalate **M)** Mono(2-ethylhexyl) phthalate.

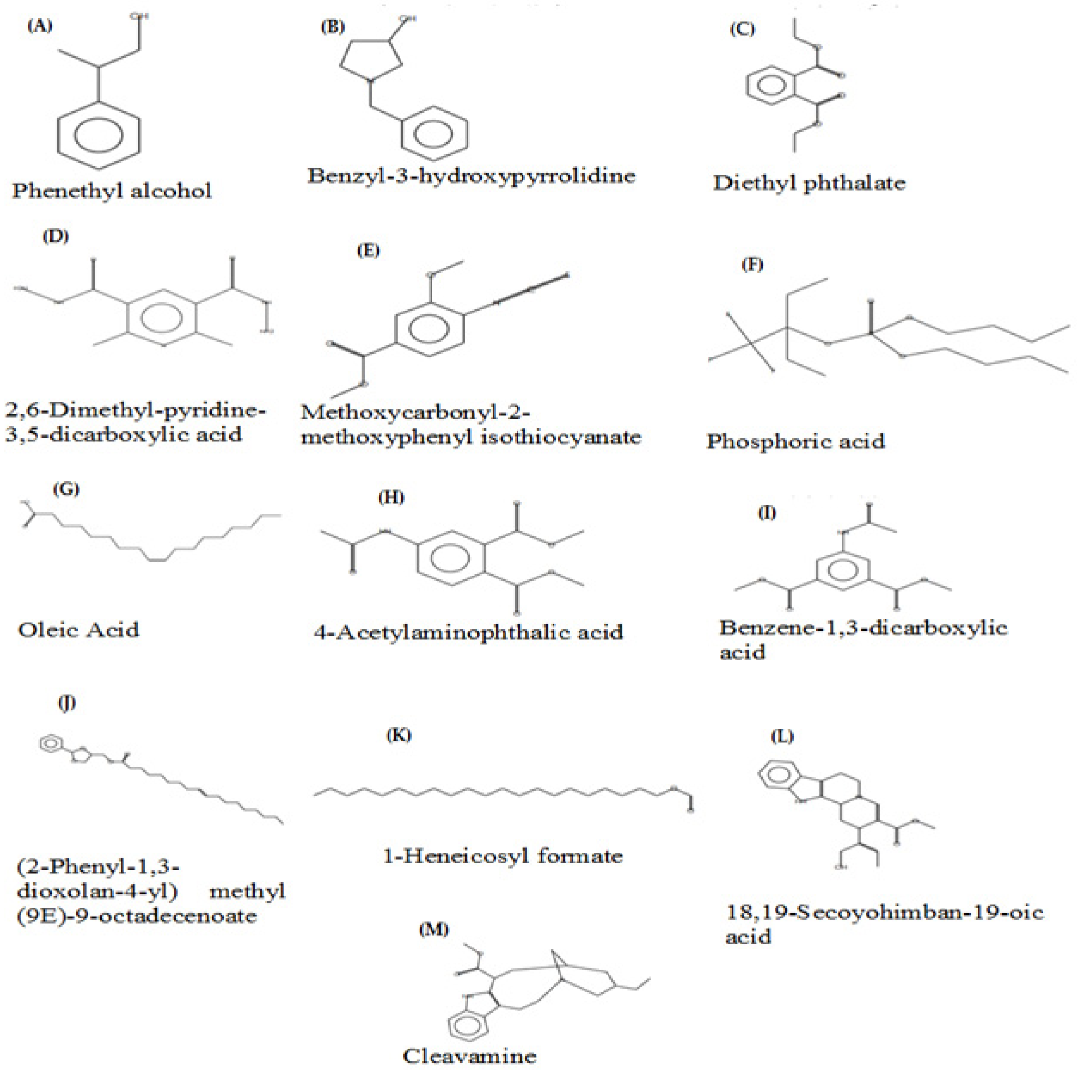

Figure S7. Major phytochemical compounds identified in fraction D of *A. articulata*.

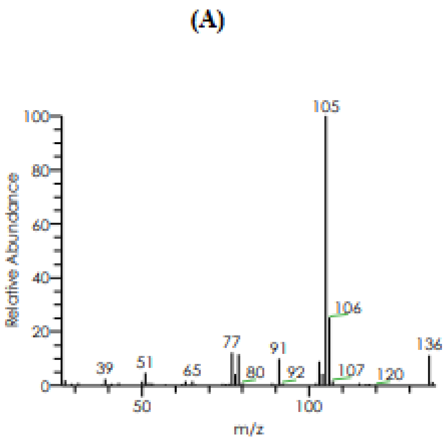

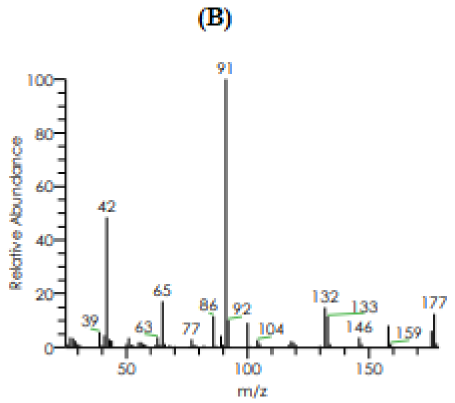

(C)

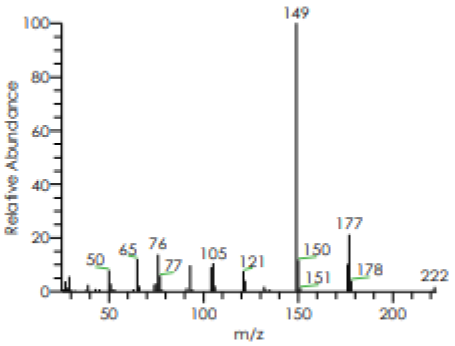
Relative Abundance
m/z
149
177
50
65
76
77
105
121
150
151
178
222

(D)

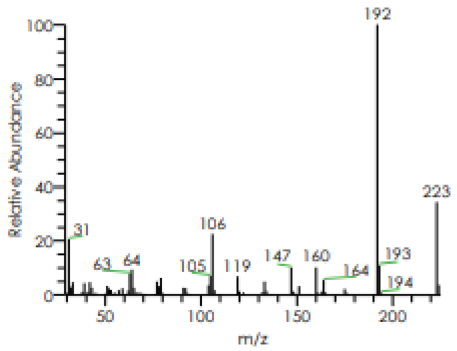
Relative Abundance
m/z
192
223
31
106
63
64
105
119
147
160
164
193
194

(E)

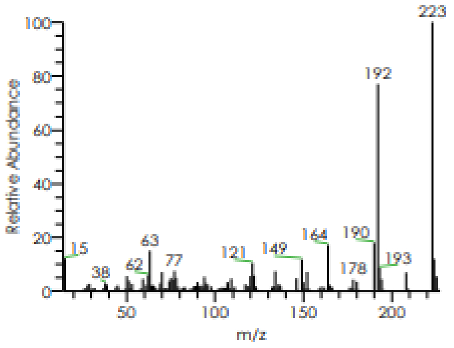
Relative Abundance
m/z
223
192
15
38
63
62
77
121
149
164
190
178
193

(F)

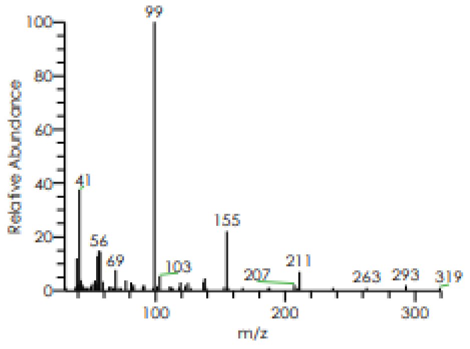
Relative Abundance
m/z
99
41
56
69
155
103
207
211
263
293
319

(G)

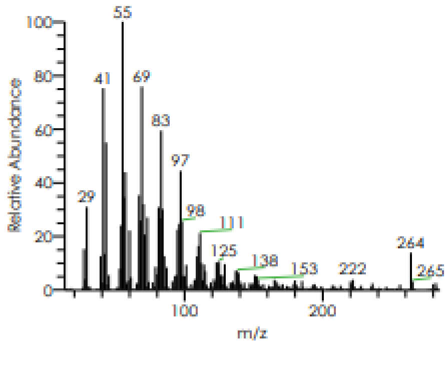
Relative Abundance
m/z
55
41
69
83
97
29
98
111
125
138
153
222
264
265

(H)

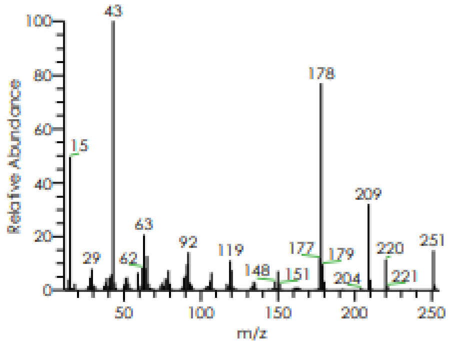
Relative Abundance
m/z
43
178
15
209
63
29
62
92
119
148
151
177
179
204
220
221
251

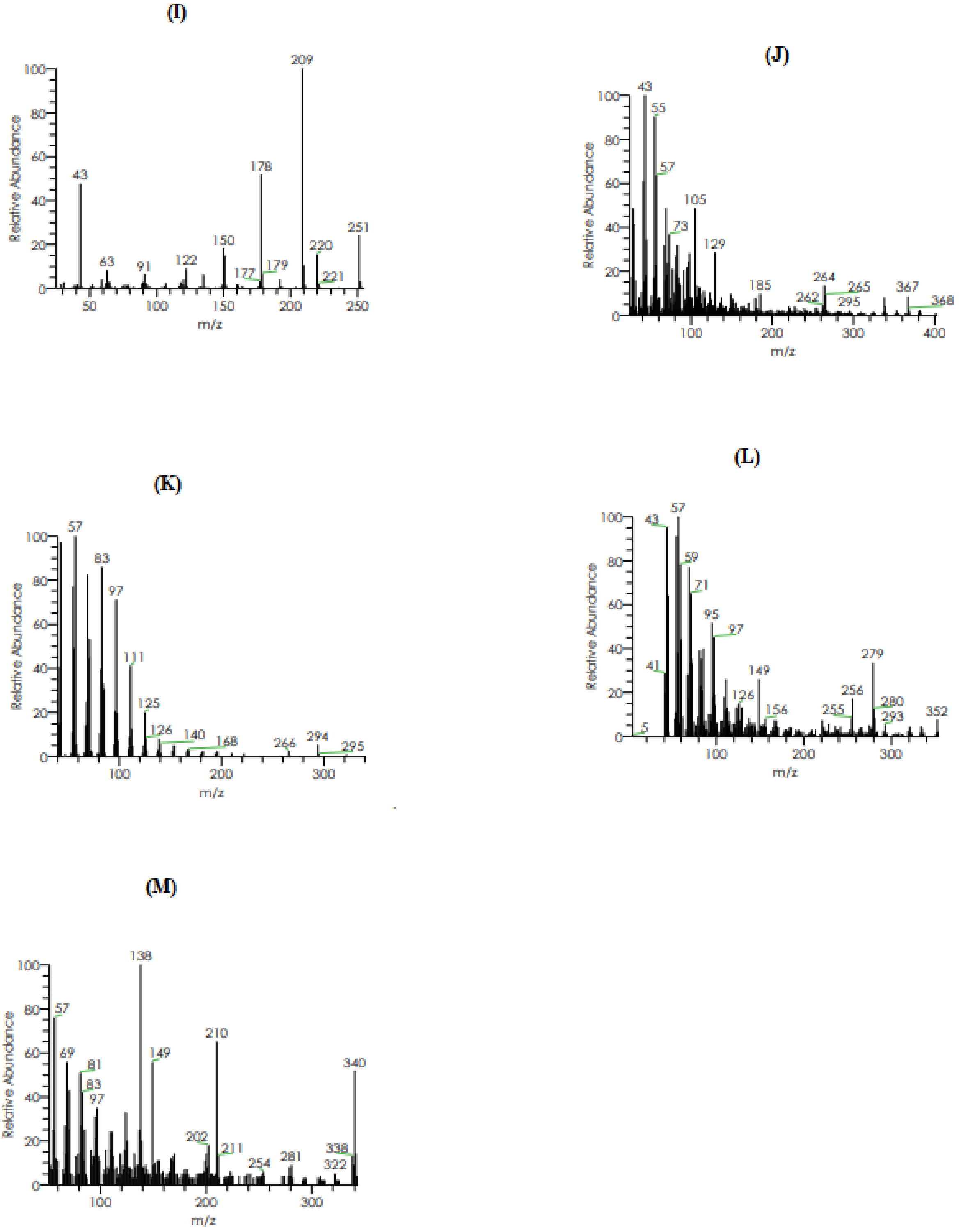

Figure S8. Individual mass fragmentation patterns of each compound: **A)** Phenethyl alcohol, á-methyl **B)** Benzyl-3-hydroxypyrrolidine **C)** Diethyl Phthalate **D)** 2,6-Dimethyl-pyridine-3,5-dicarboxylic acid, dihydrazide **E)** Methoxycarbonyl-2-methoxyphenyl isothiocyanate **F)** Phosphoric acid, dibutyl 3-trifluoromethyl-3-pentyl ester **G)** Oleic Acid **H)** 4-Acetylaminophthalic acid, dimethyl ester **I)** Benzene-1,3-dicarboxylic acid **J)** (2-Phenyl-1,3-dioxolan-4-yl) methyl (9E)-9-octadecenoate **K)** 1-Heneicosyl formate **L)** 18,19-Secoyohimban-19-oic acid **M)** Cleavamine, 18á-carboxy-3,4à-dihydro-, methyl ester.
